# WD-repeat protein WDR13 is a novel transcriptional regulator of c-Jun and modulates intestinal homeostasis in mice

**DOI:** 10.1186/s12885-017-3118-7

**Published:** 2017-02-21

**Authors:** Vijay Pratap Singh, Saritha Katta, Satish Kumar

**Affiliations:** National Facility for Transgenic and Gene Knockout Mice, CSIR-Centre for Cellular and Molecular Biology, Uppal Road, Habsiguda, Hyderabad, 500007 India

**Keywords:** JNK signalling, c-Jun, Apoptosis, Colon, Transcriptional activator

## Abstract

**Background:**

WDR13 is a member of the WD repeat protein family and is expressed in several tissues of human and mice. Previous studies in our laboratory showed that the lack of this gene in mice resulted in mild obesity, hyperinsulinemia, enhanced beta cell proliferation and protection from inflammation. However, the molecular mechanism of WDR13 action is not well understood.

**Methods:**

In the present study, we used AOM/DSS to induce colitis-mediated colorectal tumor after establishing expression of *Wdr13* gene in colon. Further, we have used human colon cancer cell lines, HT29 and COLO205, and mouse primary embryonic fibroblast to understand the molecular mechanism of WDR13 action.

**Results:**

We observed that mice lacking *Wdr13* gene have reduced number of tumors and are more susceptible to DSS-induced colon ulcers. We also show that WDR13 is a part of multi protein complex c-Jun/NCoR1/HDAC3 and it acts as a transcriptional activator of AP1 target genes in the presence of JNK signal. Consistent with in vitro data, we observed reduced expression of AP1 target genes in colon after AOM/DSS treatment in *Wdr13* knockout mice as compared to that in wild type.

**Conclusion:**

Mice lacking *Wdr13* gene showed reduced expression of AP1 target genes and protection from colitis-induced colorectal tumors.

**Electronic supplementary material:**

The online version of this article (doi:10.1186/s12885-017-3118-7) contains supplementary material, which is available to authorized users.

## Background

Cell proliferation, cell death and differentiation are basic processes of eukaryotic organisms. These processes are regulated by numerous signals including growth factors, cytokines and extracellular signals, which decide the fate between cell cycle progression and apoptosis through cytoplasmic signalling cascades to the nucleus [1–3]. The Wnt signalling pathway is one of such well-characterized signalling pathways, which is believed to be the major pathway controlling intestinal homeostasis and cancer [4] by both canonical and non-canonical routes [5]. Canonical Wnt signalling is mediated by β-catenin and transcription factor T-cell factor/lymphoid-enhancing factor (TCF/LEF) [6]. Non-canonical Wnt signalling is activated independent of β-catenin. The latter is an important pathway mediated through activation of c-Jun N-terminal kinase (JNK) [7]. JNKs are serine/threonine kinases that belong to the group of MAP kinases, which are activated by various extracellular signals [1]. As the name indicates, JNK was first identified as a kinase that phosphorylated c-Jun N-terminus [8]. Both canonical and non-canonical Wnt signalling pathways cross talk with each other and target some common genes including *c-Myc, CyclinD1, Cd44, WNTs, MMPs* and *c-Jun* [9].

The proto-oncogene, c-Jun, belongs to the AP1 group of transcription factors [10, 11]. c-Jun heterodimerizes and forms active transcription factors with Fos and ATF families of proteins [12]. AP1 activity, in part, is regulated by phosphorylation of c-Jun at serine residues 63 and 73 and threonine residues 91 and 93 by JNK [13]. c-Jun binds the co-repressor complex NCoR1/HDAC3/TBL1/TBLR1 [14] to repress AP1 target gene transcription. The presence of signal causes recruitment of ubiquitin-conjugating/19S proteasome complex to degrade the repressor complex and recruits the co-activator complex to enhance the expression of AP1 target genes [15]. c-Jun and JNKs are crucial regulators of inflammation, proliferation, apoptosis and cell migration [14, 16, 17] and are involved in malignancy of colon tissues [18].

WDR13 is a member of the WD-repeat protein family, conserved in vertebrates and expressed ubiquitously in many tissues [19–21]. A previous report from our laboratory showed that the absence of WDR13 led to enhanced pancreatic beta cell proliferation in mice [22] and the lack of this protein in a diabetic mouse model (*Leptin receptor mutant*), which has augmented JNK activity, showed reduced levels of AP1 target genes [23] and protection from inflammation. To understand the role of this protein in cell cycle and regulation of AP1 target genes, we used colitis-induced colorectal mouse model in the present study. We show that the lack of *Wdr13* gene protects mice from AOM/DSS-induced colorectal tumors. We also show that WDR13 acts as a transcriptional activator of AP1 target genes in the presence of JNK signal.

## Methods

### Animals

All mice used in this study were maintained in C57BL/6 J genetic background. Mice were housed in normal cages with corncob bedding and a regular light/dark cycle (6.00 am to 6.00 pm) and were provided with free access to food and water. Mice were euthanized by cervical dislocation. Total 33 mice were used in this study. All animal experiments were approved by the institutional animal ethics committee of CSIR-Centre for Cellular and Molecular Biology, Hyderabad, India.

### Cell culture and transfections

Primary mouse embryonic fibroblasts (MEFs) were isolated from 13.5 *dpc* mouse embryos as described previously [23]. Tails of individual embryos were used to determine genotypes at *Wdr13* locus as described earlier [22]. MEFs were grown in culture media containing 13.3 g/L DMEM, 3.7 g/L NaHCO3, 10% serum, 50 μg/ml ampicillin and 50 μg/ml streptomycin. For analysis of proliferation curve at passage 3, 5.0×10^3^ cells were seeded in 24 well plates in triplicate and cells were counted at 48 h intervals. HEK293, MCF7, HT29, COLO205 and MIN6 cells were obtained from the National Centre for Cell Science, Pune, India (purchased from American-Type Culture Collection) and were cultured in complete media as mentioned above for MEFs. The cultures were confirmed negative for mycoplasma. For transfection of primary MEFs, Lipofectamin-LTX/Plus^™^ reagent (Invitrogen) was used, whereas for other cell lines Lipofectamin 2000 (Invitrogen) was used as per the manufacturer’s instructions. In all the reporter assays, cells after transfection were cultured in DMEM media containing 10% serum for 24 h and shifted to DMEM media containing 0.5% serum with treatment for additional time as mentioned in figure legends, except for reporter assay in UV-treated cells. In the latter experiments, cells were cultured in DMEM media containing 10% serum till the termination of experiment. JNK activity was either activated with anisomycin (1 μM-Millipore) or with UV (40 J/m^2^) for additional time mentioned in the respective figures. Reporter activity was measured using either dual reporter assay (Promega) or luciferase assay (Promega) with β-gal.

### Expression constructs

pCMV-FLAG-*Wdr13* plasmid was constructed by cloning *Wdr13* cDNA at *Eco*RI and *Xba*I sites of pCMV-FLAG plasmid using primer pairs 5’–CCGGAATTCCGGATGGCCGCGGTGTGG-3’ and 5’- CCGCTCTAGATCTAGAGCAGCACAGGGTGAC-3’. FLAG-tagged deletion constructs for WDR13 protein (Fig. 5c) namely; FL-93, FL-193, FL-293, FL-393 were constructed using forward primers

- 5’-CCCGGAATTCCGGATGGAGGACTTTGAAG-3’,
- 5’-CCCGGAATTCCGGCTGGACGGCAGCATCTCCCT-3’,
- 5’-CCCGGAATTCCGGGGCAAGAAAGTGAAGGGTGG-3’,
- 5’- CCCGGAATTCCGGCTACAGCTGAAGAGAAGCTT-3’ respectively and reverse primer
- 5’-CCGCTCTAGATCTAGAGCAGCACAGGGTGAC-3’.

**Fig. 1:**
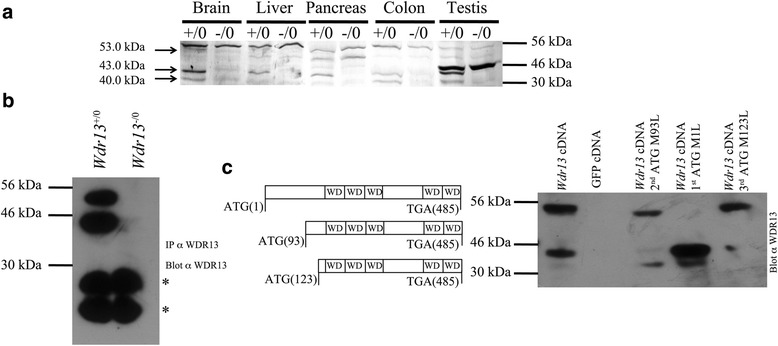
Levels of WDR13 protein isoforms in various tissues. **a** Expression of WDR13 isoforms in various tissues. **b** Immunoprecipitation from *Wdr13* ^+/0^ and *Wdr13* ^-/0^ MEFs using WDR13 antibody showed both 53.0 kDa and 43.0 kDa protein in wild type MEFs (*represents non-specific band). **c** Expression of *Wdr13* complete cDNA shows three proteins (53.0 kDa, 43.0 kDa and 40.0 kDa). Site-directed mutational studies of three ORFs (at positions 1, 93 and 123) show the absence of respective proteins

pCMV-Myc-*Wdr13* plasmid was constructed by cloning *Wdr13* cDNA at *Eco*RI and *Xba*I sites of pCMV-Myc vector containing Myc peptide sequence at the N-terminal. Myc tagged *c-Jun* over-expression vector was constructed by cloning c-Jun coding sequences at end-filled *Eco*RI and *Xba*I sites of pCMV-Myc using primer pair 5’-CCCGGAATTCCGGATGACTGCAAAGATGGAAAC-3’ and 5’- CCGCTCTAGATCTAGATCAAAACGTTTGCAACTGCT-3’. To obtain the c-Jun deletion constructs (Fig. 5d), forward primers 5’-CATGACTGCAAAGATGGAAAC-3’, 5’-CAAGAACGTGACCGACGAGCA-3’, 5’-CGCGGTGGCCTCAGTAGCAGG-3’ and reverse primers 5’-TCAAAACGTTTGCAACTGCT-3’, 5’-TCAGATCCGCTCCTGAGACT-3’ were used to amplify the respective constructs and cloned at end-filled *Eco*RI and *Xba*I sites of pCMV-Myc. All the FLAG-tagged HDAC vectors were a kind gift from Ronald M. Evans. To study the WDR13 protein isoforms, complete *Wdr13* cDNA was cloned in pCI vector (Promega). All the three predicted initiation codon ATGs (at positions 1, 93 and 123) (Fig. 1c) were mutated to CTG using phusion site directed mutagenesis kit (F541-NEB). The primers used for SDM were the following-

- 1^st^ ATG FP- 5’-AGAAGGAAGCCAGGGACTGGCCGCGGTGTGGCA-3’
- 1^st^ ATG RP- 5’-TGCCACACCGCGGCCAGTCCCTGGCTTCCTTCT-3’
- 93^rd^ ATG FP- 5’- AACAACCCTTGATCGACTGGAGGACTTTGAAGA-3’
- 93^rd^ ATG RP- 5’-TCTTCAAAGTCCTCCAGTCGATCAAGGGTTGTT-3’
- 123^rd^ ATG FP –5’-CCAGCTGCAGGCACAACTGAACCGTGCAGTCTA-3’
- 123^rd^ ATG RP- 5’-TAGACTGCACGGTTCAGTTGTGCCTGCAGCTGG-3’

PCR reactions were performed using phusion hot start high fidelity DNA polymerase (F540-NEB), and the amplicons were confirmed by DNA sequencing.

### RNA isolation, reverse transcription, real time PCR and western blot analysis

Total RNA was isolated using RNeasy Mini Kit (Qiagen). Reverse transcription was performed using ImProm-IITM kit (Promega) after DNase (Promega) treatment of RNA samples. Real time PCR was performed for various genes using Syber Green master mix (Invitrogen) and the list of primers sequences is provided in Additional file 1: Table S1. Western blots were performed after extraction of proteins from various tissues and cell lines in RIPA buffer and blotted on PVDF membrane. Anti-WDR13 purified antibody (HPA000913), FLAG (F3165) from Sigma, p53 (sc-126), c-Jun (sc-45), p-c-Jun (sc-822), SMRT (sc-1610), actin (sc-47778) from Santacruz, p-JNK (9251), t-JNK (9252), NcoR1 (5948) from Cell Signalling and Myc-HRP (R951-25) from Invitrogen were used for visualization of the respective proteins.

### Cell cycle analysis and apoptosis

MEFs were cultured in complete media till passage 2. At this stage, cells were seeded in 100 mm dish at a density of 1×10^6^ cells per dish. To unsynchronized cells 10 μM BrdU was added for 30 min and cells were collected by trypsinization. Cells were washed with PBS, fixed in 70% ethanol and stored at −20 °C. BrdU FITC Set (556028, BD Pharmingen™) was used to stain BrdU-positive cells. Cells were analyzed using fluorescence activated cell sorter (BD FACS Calibur). Annexin V – FLUOS (1828681-Roche) was used to study the number of apoptotic cells using BD FACS Calibur.

### Immunoprecipitation

For co-immunoprecipitation, DNAs were transfected into HEK293 cells using Xfect reagent. After 48 h, cells were lysed in lysis buffer (50 mM –Tris.HCl, 150 mM-NaCl, 1 mM-EDTA, 1% Triton X-100 and protease inhibitor cocktail). Cell lysate was centrifuged and pre-cleaned with Protein G. Anti-FLAG agarose beads (F2220-Sigma) were added to the pre-cleaned lysate and incubated for 4 h at 4 °C. Immuno complex was washed 4x with wash buffer (50 mM –Tris.HCl, 150 mM-NaCl, 1 mM-EDTA, 0.5%-Triton X-100 and Protease inhibitor cocktail). The immunocomplex was eluted, separated on 6–10% SDS PAGE and blotted on PVDF membrane. For endogenous protein-protein interactions, MEFs were collected from T150 for each immunoprecipitation reaction and lysed in lysis buffer as mentioned above. Cell lysates were pre-cleaned with Protein A, and 4 μg of αWDR13 antibody was used for immunoprecipitation.

### Chromatin immunoprecipitation

MEFs were grown in complete DMEM media in T150 for each ChIP experiment. The cells were crosslinked in 1% formaldehyde for 10 min at room temperature and scraped in PBS containing protease inhibitors. Chromatin immunoprecipitation was performed using chromatin immunoprecipitation (ChIP) Assay Kit (Millipore 7–295) as per the manufacturer’s instructions. Briefly, cells were lysed in SDS lysis buffer and sonicated for 42 cycles (30 s on/30 s off) using Bioruptor (Diagenode) to get ~200–500 bp product size. The cell lysate was pre-cleaned by incubating in agarose beads for 1 h at room temperature, followed by immunoprecipitation with 3 μg anti-WDR13. The beads were washed and the genomic DNA fragments were eluted and purified using phenol/chloroform extraction for real time PCR. To obtain an amplicon spanning the AP1 site, 5’-CATTACCTCATCCCGTGAGC-3’ and 5’- ATCCAGCCTGAGCTCAACAC-3’ primer pair was used.

### AOM/DSS model of colon carcinoma

To induce colon carcinoma, mice were injected intraperitoneally with 10 mg/kg body weight of AOM (Azoxymethane-Sigma) dissolved in saline [24]. Seven days after AOM injection, 2% DSS (Dextran sodium sulphate -Sigma) was given in drinking water for the next seven days, followed by normal water until the 21^st^ week. At the 21^st^ week, mice were sacrificed after 1.5 h of BrdU injection (100 mg/kg body weight). For macroscopic examinations, colons were opened longitudinally and fixed overnight in buffered 4% para-formaldehyde. For histological examination, colons were fixed overnight in buffered 4% para-formaldehyde, rolled, embedded in paraffin and sectioned (4 μm thickness). Sections were mounted on positively charged slides (Fisher Scientific) and were stained either with H&E or with anti-BrdU antibodies. Primary antibody was detected with superpicture^™^ kit (87-8963- Invitrogen). For apoptosis, TUNEL assay was performed as per the manufacturer’s instructions (Promega). For western blot and real time PCR, proximal colon was collected in PBS, snap frozen in liquid nitrogen and stored at −80°C.

### DSS treatment to induce colon injury

Two percent DSS was given in drinking water for 7 days to mice at 2 months of age. On day 7, DSS was replaced with normal drinking water, which was continued till the 10^th^ day. On the 10^th^ day mice were sacrificed after 1.5 h of BrdU injection (100 mg/kg body weight). For histological examination, colons were fixed overnight in buffered 4% para formaldehyde and processed as mentioned above.

### Statistical analysis

The unpaired two-tailed student’s *t*-test was used for statistical analysis. Microsoft Excel software was used for calculation of *P* values. Data are presented as mean ± s.e.m.

## Results

### Identification of WDR13 protein isoforms and their differential expression

Previous results from our laboratory showed that *Wdr13* mRNA is present in various tissues of human and mouse [19, 20], and using *Wdr13* knockout mice we had earlier analysed the role of this gene in beta cells [22]. However, the role of this protein in other tissues is not known. First, we performed western blot analysis to examine the expression of this protein in various tissues. Various tissues showed the presence of different isoforms of WDR13 protein. The tissues examined such as liver, pancreas, colon and testis had 43.0 kDa and 40 kDa protein isoforms (Fig. 1a). Interestingly, brain tissues showed an additional isoform of 53.0 kDa (Fig. 1a). Since we observed some non-specific bands in western blots using anti WDR13 antibody, we derived primary embryonic fibroblasts from *Wdr13* ^+/0^ and *Wdr13* ^-/0^ mouse embryos at 13.5 *dpc* [23] and performed immunoprecipitation to validate the antibody. Consistent with the genotypes of the cells, we found 53.0 kDa and 43.0 kDa protein isoforms from *Wdr13* ^+/0^ MEFs and these were absent in *Wdr13* ^-/0^ MEFs (Fig. 1b). *Wdr13* gene contains three ATGs in the predicted open reading frame at positions 1, 93 and 123. Therefore, to rule out the possibility that smaller isoforms were the cleavage products of the larger protein, we cloned complete *Wdr13* cDNA in pCI mammalian expression vector (Promega) and mutated all the three ATGs (M) to CTG (L) by site-directed mutagenesis. *Wdr13* over-expression of complete cDNA in HEK293 cells showed all the three isoforms (Fig. 1c). However, mutation of the respective ATGs led to the loss of expression of the corresponding isoforms (Fig. 1c). These results confirmed that the different isoforms of WDR13 were not cleavage products of the large isoform but were derived from three different translation initiation codons.

### Absence of *Wdr13* gene greatly reduces the incidence of colitis-induced colorectal tumor

**Fig. 2:**
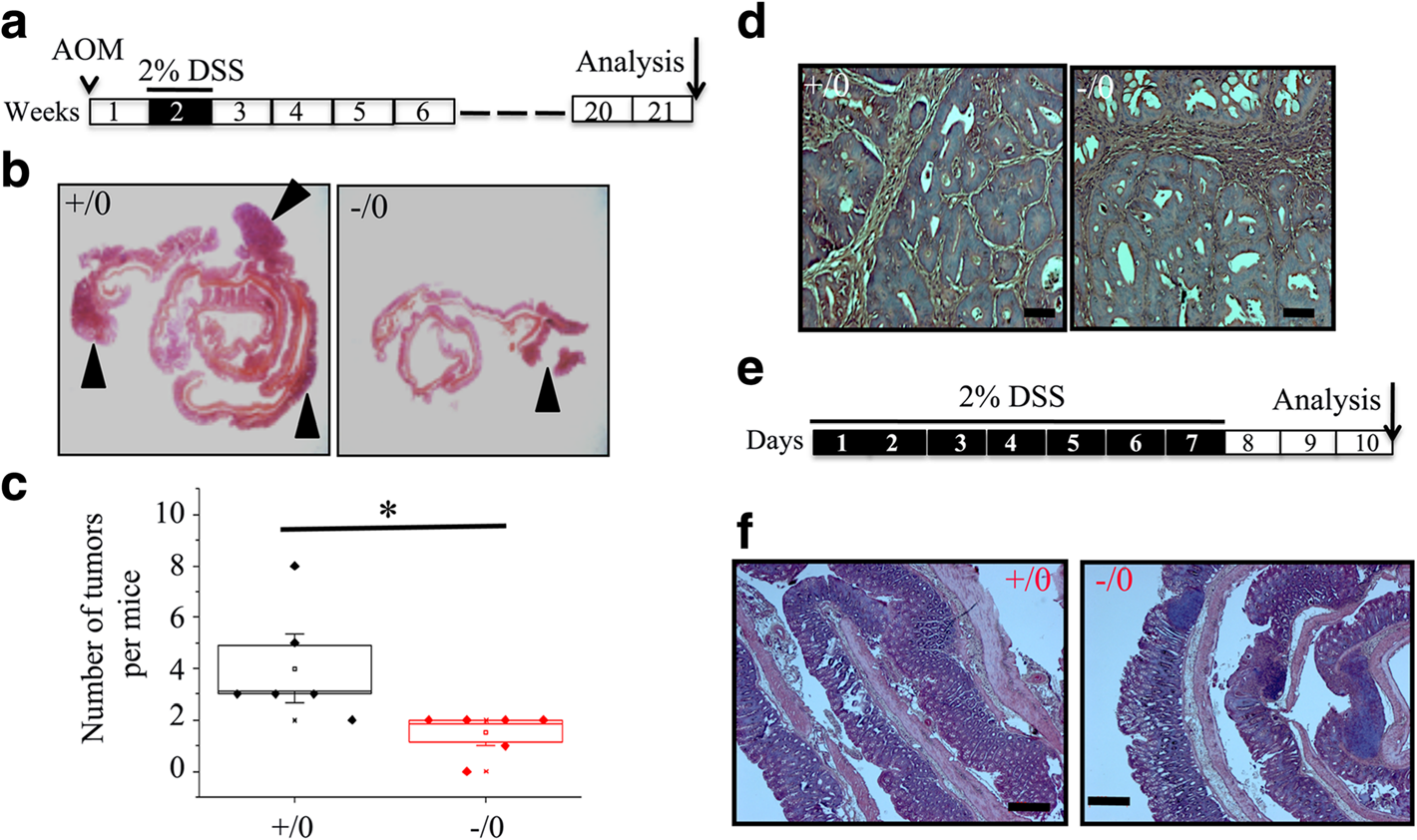
*Wdr13* accelerates colitis-induced colon tumor. **a** Schematic representation of the experimental design for colitis-induced colon tumor with azoxymethane. **b** H&E staining of colon from *Wdr13* ^+/0^ and *Wdr13* ^-/0^ mice. **c** Quantification of number of tumors after AOM/DSS treatment (*n* = five *Wdr13* ^+/0^ and six *Wdr13* ^-/0^ mice). **d** H&E staining of tumor morphology from *Wdr13* ^+/0^ and *Wdr13* ^-/0^ mice. **e** Schematic representation of the experimental design for DSS-induced intestinal regeneration (*n* = four *Wdr13* ^+/0^ and four *Wdr13* ^-/0^ mice). **f** H&E staining of colon from *Wdr13* ^+/0^ and *Wdr13* ^-/0^ mice after DSS treatment

Since the WDR13 protein isoforms are present in various tissues and it is a negative regulator of beta cell proliferation [22], we observed these knockout mice till one year of age for any tumor progression. We did not observe any spontaneous tumor development until one year of age. Given the presence of this protein in colon, we used AOM/DSS (Mr- 40,000) to induce colorectal tumor in order to know whether the lack of *Wdr13* gene affected tumor progression. The schematic protocol is given in Fig. 2a. We did not find any significant difference in the body weight till the termination of experiments (21^st^ week after AOM injection). Macroscopic examination of colon from the wild-type mice showed 3–7 tumors in distal and middle colon in all animals as expected. Surprisingly, contrary to our hypothesis, *Wdr13* ^*-/0*^ mice had only 0–2 tumors each in their distal colon (Fig. 2b, c). Histological examination of tumor showed adenocarcinoma (Fig. 2d). It is known that DSS treatment causes ulceration and colitis in colon. To rule out the possibility of resistance to DSS-induced ulceration and colitis in *Wdr13* ^-/0^ mice, we analysed colon histology of *Wdr13* ^*+/0*^ and *Wdr13* ^*-/0*^ mice during the recovery period (Fig. 2e). Histological examination of colon from *Wdr13* ^*+/0*^ and *Wdr13* ^*-/0*^ mice showed mucosal ulceration and acute inflammatory infiltration (Fig. 2f). Interestingly, *Wdr13* ^*-/0*^ mice showed more ulceration as compared to *Wdr13* ^*+/0*^ mice, suggesting their enhanced susceptibility to DSS treatment (Fig. 2f).

### *Wdr13*^*-/0*^ mice showed increased apoptosis after AOM/DSS-induced colitis

**Fig. 3:**
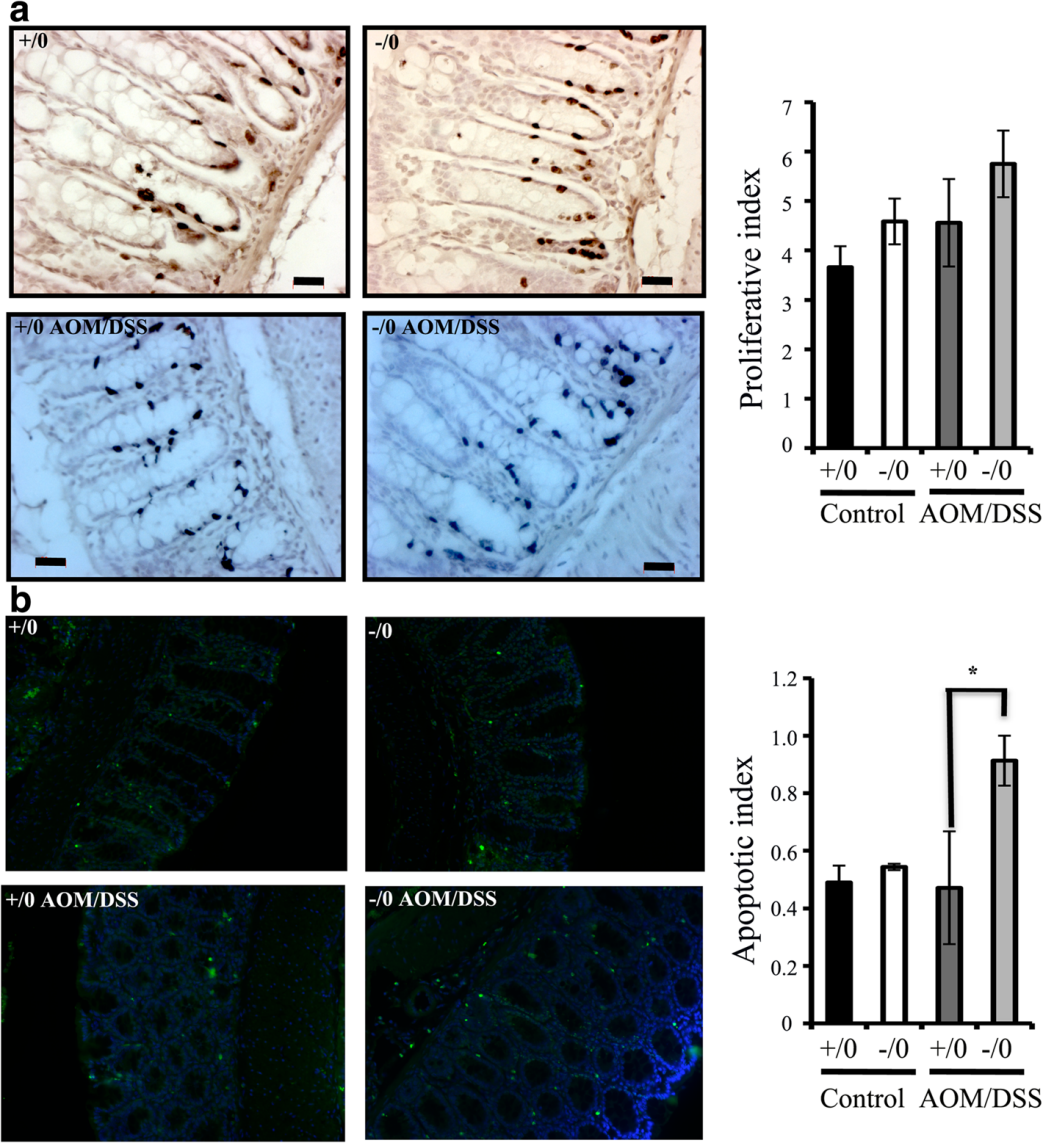
Cell proliferation and TUNEL analysis of *Wdr13* ^+/0^ and *Wdr13* ^-/0^ mice colon. **a** BrdU immunohistochemistry for BrdU positive cells on representative colonic crypts of mice with indicated genotype and treatment. Black bar, *Wdr13* ^+/0^ mice (*n* = 34); white bar, *Wdr13* ^-/0^ mice (*n* = 31); Dark grey bar, AOM/DSS-treated *Wdr13* ^+/0^ mice (*n* = 28); Light grey bar, AOM/DSS-treated *Wdr13* ^-/0^ mice (*n* = 31). **b** TUNEL staining for apoptosis on representative colonic crypts of mice with indicated genotype and treatment. Black bar, *Wdr13* ^+/0^ mice (*n* = 132); white bar, *Wdr13* ^-/0^ mice (*n* = 162); Dark grey bar, AOM/DSS-treated *Wdr13* ^+/0^ mice (*n* = 121); Light grey bar, AOM/DSS-treated *Wdr13* ^-/0^ mice (*n* = 93). n- Shows number of crypts counted for respective genotype. Scale bar, 20 μm, *- *p* <0.05

To understand the mechanism of protection from colitis-induced colon tumor in *Wdr13* ^*-/0*^ mice, we measured the proliferative and apoptotic index of control, and AOM/DSS-treated *Wdr13* ^*+/0*^ and *Wdr13* ^*-/0*^ mice. The proliferative index was measured by counting the number of BrdU positive cells per crypt excluding tumors. There were no significant differences in proliferative index between *Wdr13* ^*+/0*^ and *Wdr13* ^*-/0*^ mice (Fig. 3a). Further, we measured the apoptotic cells per crypt excluding tumors using TUNEL assay. Apoptotic index between the untreated *Wdr13* ^*+/0*^ and *Wdr13* ^*-/0*^ mice was similar. However, after AOM/DSS-induced colitis, there was a significant increase in the number of apoptotic cells in *Wdr13* ^*-/0*^ mice as compared to *Wdr13* ^*+/0*^ mice (Fig. 3b). These results suggest that the increased apoptosis may be responsible for the removal of cells mutated by AOM, and thus for lower tumor burden in *Wdr13* deficient mice.

### WDR13 is a transcriptional regulator of c-Jun/AP1 target genes

**Fig. 4:**
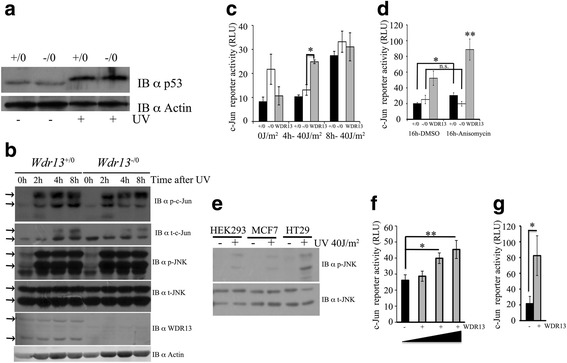
WDR13 activates c-Jun activity in JNK-dependent manner. **a** Levels of p53 in *Wdr13* ^+/0^ and *Wdr13* ^-/0^ MEFs before and 24 h after UV treatment (40 J/m^2^). Actin was used as loading control. **b** Effect of UV on the expression of p-c-Jun, t-c-Jun, p-JNK, t-JNK and WDR13 in *Wdr13* ^+/0^ and *Wdr13* ^-/0^ MEFs. Actin was used as loading control. Arrows show specific band. **c** c-Jun promoter-reporter activity in UV untreated and treated (40 J/m^2^) *Wdr13* ^+/0^, *Wdr13* ^-/0^ and MEFs – over-expressing WDR13 (c-Jun-Luc- 350 ng, SV40-RL-100 ng, pGFP-150 ng or pCMV-FLAG-*Wdr13-*150 ng). **d** c-Jun promoter-reporter activity in anisomysin untreated and treated (16 h) *Wdr13* ^+/0^, *Wdr13* ^-/0^ and MEFs – over-expressing WDR13 (c-Jun-Luc- 350 ng, β-gal-100 ng, pGFP-150 ng or pCMV-FLAG-*Wdr13-*150 ng). For panel C and D, Black bar, *Wdr13* ^+/0^ MEFs; white bar, *Wdr13* ^-/0^ MEFs; Light grey bar, MEFs over-expressing WDR13;. **e** p-JNK and t-JNK levels in HEK293, MCF7 and HT29 cells before UV treatment and 2 h after UV treatment. **f** c-Jun promoter-reporter activity in HT29 cells shows activation of reporter after *Wdr13* over-expression in dose-dependent manner (c-Jun-Luc-250 ng, SV40-RL-100 ng, pGFP-250 ng, 200 ng, 150 ng, 100 ng, and pCMV-FLAG-*Wdr13-*0 ng, 50 ng, 100 ng, 150 ng). **g** c-Jun promoter-reporter activity in COLO205 cells shows activation of reporter after *Wdr13* over-expression (c-Jun-Luc-350 ng, SV40-RL-50 ng and pGFP-200 ng or pCMV-FLAG-*Wdr13-*200 ng). All transfections were performed in technical triplicates. Student’s *t-*test was used for statistical analysis and error bar shows s.e.m

To understand the mechanism of WDR13 action, we characterized the growth properties of *Wdr13* ^*+/0*^ and *Wdr13* ^*-/0*^ MEFs [23]. During initial passages, cell growth, cell proliferation and apoptosis were similar in *Wdr13* ^*+/0*^ and *Wdr13* ^*-/0*^ MEFs (Additional file 2: Figure S1). Previous study in our laboratory showed that the over-expression of *Wdr13* up-regulated p21 protein levels, whereas *Wdr13* knockout mice showed down-regulation of p21 protein levels in pancreatic islets [22]. Consistent with these results, p21 promoter reporter in beta cells (MIN6) showed activation of reporter activity upon WDR13 over-expression (Additional file 3: Figure S2C). p21 protein level is regulated at the transcription and post transcription/post translation levels by various transcription factors, of which p53 *and* c-Jun are well documented [25, 26]. Therefore, we analysed the expression of p53 and c-Jun protein levels in *Wdr13* ^*+/0*^ and *Wdr13* ^*-/0*^ MEFs before and after UV treatment. There was no difference in *p53* expression levels between the two genotypes (Fig. 4a). As expected, after UV treatment *Wdr13* ^*+/0*^ MEFs showed increase in c-Jun protein levels with time. However, *Wdr13* ^*-/0*^ MEFs did not show any increase in c-Jun protein levels (Fig. 4b). The basal level of c-Jun protein before UV treatment in *Wdr13* ^*-/0*^ was higher than that in *Wdr13* ^*+/0*^ MEFs. The reason of this difference is not clear. c-Jun binds at AP1 sites in the genome and regulates transcription of many genes including its own [13]. The phosphorylation of c-Jun by JNK leads to activation of c-Jun [15, 27], and it is well established that UV-treatment activates JNK pathway by increasing the phosphorylation of JNK [28]. As expected, there was a significant increase in p-JNK levels after UV-treatment in both *Wdr13* ^*+/0*^ and *Wdr13* ^*-/0*^ MEFs (Fig. 4b).

To further confirm the immunoblot results and to understand the role of WDR13 in c-Jun transcriptional regulation, we used c-Jun reporter containing multiple (3X) AP1 sites. Consistent with immunoblot results, 8 h after UV-treatment *Wdr13* ^+/0^ MEFs, *Wdr13* ^-/0^ MEFs and MEFs over-expressing WDR13 showed 3.3-, 1.5- and 3.6-fold induction in the reporter activity (Fig. 4c). Similar to UV-induction, activation of JNK activity by anisomycin also activated c-Jun reporter activity ≈1.8 fold in *Wdr13* ^+/0^ MEFs and MEFs over-expressing WDR13. However, *Wdr13* ^-/0^ MEFs did not show any activation in reporter activity (Fig. 4d). Consistent with immunoblot results, we also observed increased AP1 reporter activity in *Wdr13* ^*-/0*^ as compared to that in *Wdr13* ^*+/0*^ MEFs before JNK activation either by UV or by anisomycin. Next, we performed c-Jun reporter assay in human colon cancer cell line HT29 that has high p-JNK activity (Fig. 4e). Over-expression of *Wdr13* gene in HT29 showed a dose-dependent activation of c-Jun reporter (Fig. 4f). Similar results were obtained in another colon cancer cell line, COLO205 (Fig. 4g). Also, activation of JNK activity in MIN6 and HEK293 cells by UV-treatment showed activation of c-Jun reporter after WDR13 over-expression (Additional file 3: Figure S2B, D). These results suggest that WDR13 activates AP1 target genes in the presence of JNK activity.

### WDR13 interacts with c-Jun

**Fig. 5:**
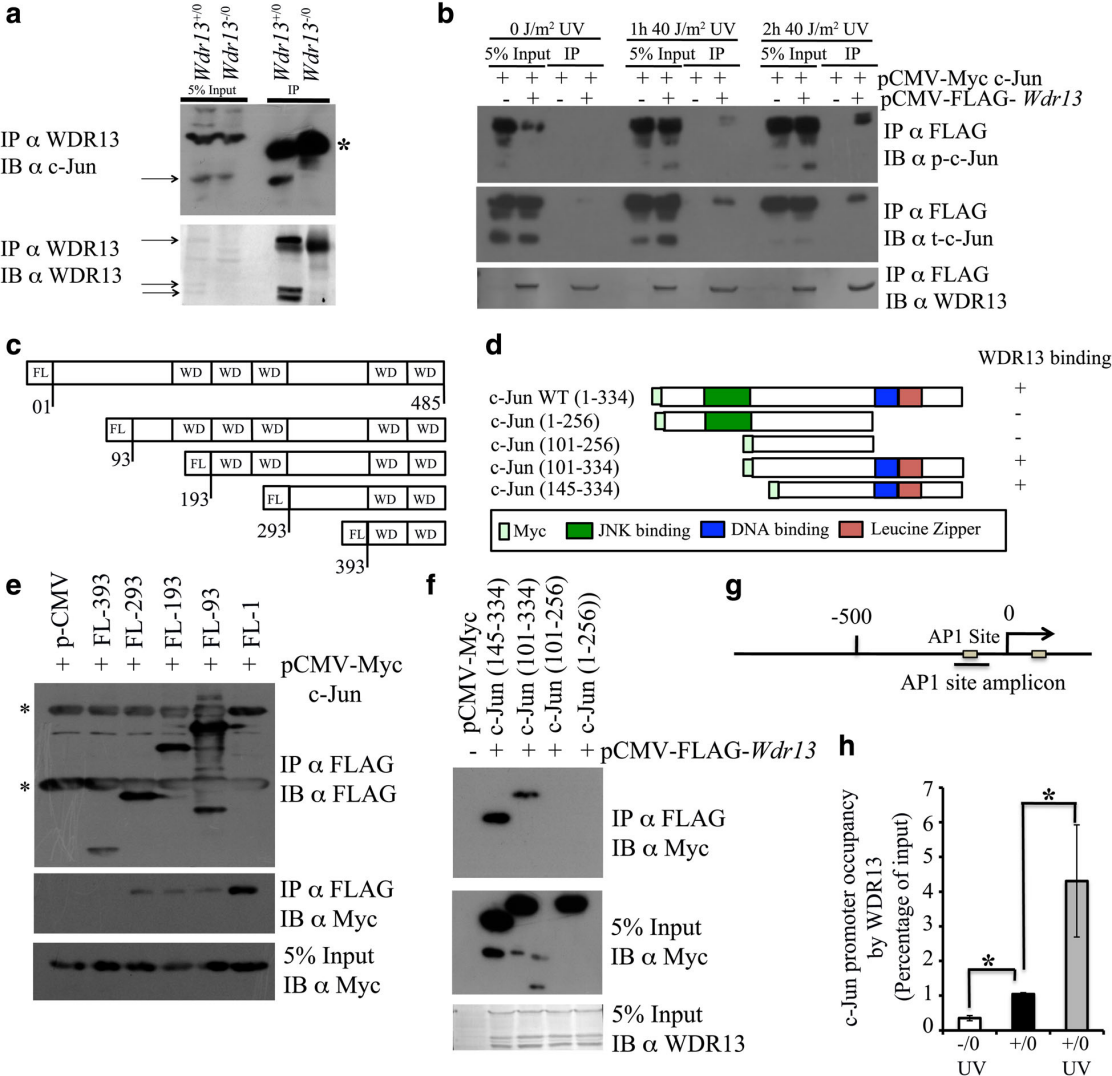
WDR13 interacts with c-Jun and binds at AP1 sites of c-Jun promoter. **a** Immunoprecipitation, using anti WDR13 antibody from mouse embryonic fibroblasts shows interaction of WDR13 with c-Jun. *Wdr13* knockout MEFs were used as control. Arrows show specific band. * Denotes non-specific band in input and IgG band in IP samples.**b** Co-immunoprecipitation of Myc-c-Jun and FLAG-WDR13 using anti FLAG agarose beads in HEK293 cells show interaction of WDR13 with c-Jun. Interaction of WDR13 with c-Jun is enhanced as phosphorylation status of c-Jun increases. **c** Domain organization of WDR13 and schematic of various deletion constructs. **d** Domain organization of c-Jun and schematic of various deletion constructs. **e** Co-immunoprecipitation of Myc-c-Jun and various deleted FLAG-tagged WDR13 domains using anti FLAG agarose beads in HEK293 cells shows that region of 293–393 amino acids is essential for interaction. 5% input shows protein expression in cell lysate with Myc specific antibody in the lower panels. **f** Co-immunoprecipitation of FLAG-*Wdr13* and various deleted Myc tagged c-Jun domains using anti FLAG agarose beads in HEK293 cells shows interaction of WDR13 with DNAdimerization and DNA-binding domain of c-Jun. 5% input shows protein expression in cell lysate with WDR13-specific antibody in the lower panels. **g** Schematic overview of *c-jun* promoter. Primer pairs used for ChIP from AP1 site were underlined. **h** WDR13 binding to AP1 site of c-Jun promoter in MEFs. *Wdr13* knockout MEFs was used as ChIP control. *White bar*, *Wdr13* ^-/0^ MEFs 2 h after UV treatment; *black bar*, *Wdr13* ^+/0^ MEFs; *Light grey bar*, *Wdr13* ^+/0^ MEFs 2 h after UV treatment. ChIP experiment was performed from independently derived two MEFs line for each genotype. Student’s *t-*test was used for statistical analysis and error bar shows s.e.m

Having established that WDR13 is involved in transcriptional regulation of c-Jun and given that the latter autoregulates its own promoter, we first analysed the interaction of WDR13 with c-Jun. Using anti WDR13 antibody, we purified the immuno-complex from *Wdr13* ^+/0^ MEFs. Immuno-blotting of the immuno-complex showed interaction of WDR13 with c-Jun (Fig. 5a). To understand the effect of phosphorylation state of c-Jun on its interaction with WDR13, Myc-c-Jun and FLAG-*Wdr13* were over-expressed in HEK293 cells, and 48 h after transfection, cells were subjected to UV-treatment to activate JNK. Interestingly, the interaction of WDR13 is enhanced by c-Jun phosphorylation status (Fig. 5b). Domain mapping showed that amino acids 293 to 393 of WDR13 (Fig. 5c, e) and DNA-binding and dimerization domains of c-Jun (Fig. 5d, f) are important for interaction between c-Jun and WDR13. Further, we analysed the interaction of WDR13 with c-Jun promoter using ChIP in MEFs. The results showed occupancy of WDR13 at AP1 site of c-Jun promoter (Fig. 5g, h). Interestingly, occupancy of WDR13 increases ≈ 4 fold at AP1 site of c-Jun promoter following JNK activation.

### WDR13 is a part of c-Jun/NCoR1/HDAC3 complex

**Fig. 6:**
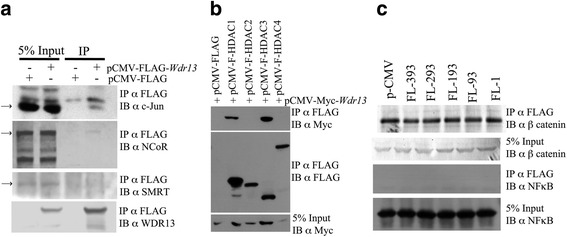
Interactome of WDR13. **a** Interaction of FLAG-tagged WDR13 with endogenous NCoR1, SMRT and c-Jun in HEK293 cells. WDR13 interacts with endogenous NCoR1, c-Jun but not with SMRT. Arrows show specific band. **b** Co-immunoprecipitation of Myc-*Wdr13* and various HDACs show interaction of WDR13 with HDAC1 and HDAC3 in HEK293 cells. 5% input shows protein expression in cell lysate with Myc-specific antibody in the lower panels. **c** Interaction of β-catenin and NFκB with various deletion constructs of FLAG-*Wdr13* shows absence of evidence of any interactions in HEK293 cells. Domain organization of WDR13 and schematic of various deletion constructs used in these immunoprecipitation experiments are mentioned in Fig. 5c

Having established that WDR13 is involved in transcriptional regulation and it interacts with c-Jun, we proceeded to identify other possible interacting partners of this protein by purifying the WDR13-containing immunocomplex from HEK293 cells over-expressing FLAG-tagged WDR13. Consistent with our above results, we observed that WDR13 interacts with c-Jun (Fig. 6a). In search of co-repressors and co-activators of c-Jun present in the immunocomplex, western blot was performed for various proteins. We observed that WDR13 interacts with NCoR1 but found no evidence of its interaction with SMRT (Fig. 6a). Among the various HDACs analysed, interaction of WDR13 was observed with HDAC1 and HDAC3 (Fig. 6b). We did not find any evidence of interaction of WDR13 with β-catenin and NFκB (Fig. 6c). Taken together, these data showed that WDR13 regulates AP1 activity by interacting with NCoR1/HDAC3 complex but not with SMRT/HDACs.

### WDR13 regulates AP1 target genes in colon

**Fig. 7:**
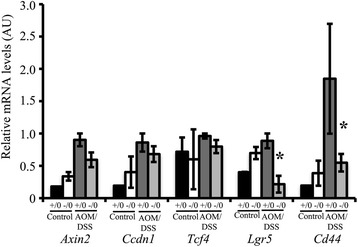
WDR13 regulates c-Jun function in vivo. Expression analysis of AP1 target genes from proximal colon of indicated genotypes and treatments (excluding tumors). Expression levels were normalized to *gapdh*. Black bar, *Wdr13* ^+/0^ mice (*n* = 2); white bar, *Wdr13* ^-/0^ mice (*n* = 2); Dark grey bar, AOM/DSS-treated *Wdr13* ^+/0^ mice (*n* = 2); Light grey bar, AOM/DSS-treated *Wdr13* ^-/0^ mice (*n* = 3). n- Shows number of mice used in respective genotype. All real time PCR was performed in technical triplicate. Student’s *t-*test was used for statistical analysis and error bar shows s.e.m

Since our in-vitro experiments showed that WDR13 regulates AP1 target genes, and as the AOM/DSS-cancer model is known to have higher expression of AP1 target genes, we studied the expression of these genes in colon. First, we analysed the expression of various AP1 target genes that are known to have role(s) in cell proliferation and apoptosis in proximal colon. We observed increased expression of AP1 target genes in the colon of AOM/DSS-treated control mice (Fig. 7). Interestingly, there was significant reduction in the levels of *lgr5* and *cd44* transcripts in *Wdr13* ^*-/0*^ mice as compared to those in *Wdr13* ^*+/0*^ mice after AOM/DSS treatment (Fig. 7). These results support the role of WDR13 in regulation of AP1 target genes under JNK activation.

## Discussion

In the present study, we have shown that *Wdr13* knockout mice were protected to a large extent from AOM/DSS-induced colorectal tumor. The primary reason for the protection against colitis-induced colorectal tumor in *Wdr13* knockout mice is likely to be the resultant ulceration and the increased apoptosis. Experiments designed to co-purify the immune-complex containing WDR13 led to the identification of c-Jun and NCoR1/HDAC3 complex as interacting partners of WDR13. We have also shown that WDR13 acts as a transcriptional activator of AP1 target genes in the presence of JNK signal.

Repression of c-Jun occurs through various repressor complexes, namely; Mbd3/NuRD [29], Fbl10/Sin3a/HDACs [30], NCoR1/HDAC3 [14, 27, 31] and SMRT/HDACs [32]. The existence of many repressors for a single transcription factor such as c-Jun may be required for its tightly controlled regulation in various physiological conditions. Auto regulation of c-Jun further explains the necessity of this tight regulation. From our in-vivo and in-vitro results, it is evident that WDR13 regulates c-Jun transcription and activates AP1 transcription after activation of JNK. Evidence of physical interaction of WDR13 with c-Jun/NCoR1/HDAC3 (Fig. 6a), and the increased WDR13 occupancy at c-Jun promoter after activation of JNKs (Fig. 5h) suggest an important role of this protein in c-Jun transcriptional regulation. The presence of WDR13 in NCoR1/HDAC3 complex and its interaction with c-Jun raise some interesting questions-1) How does WDR13 activate AP1 target genes in the presence of JNK signal? 2) Does this protein have a role in proteosomal degradation of NCoR1/HDAC3 and exchange of co-repressors with co-activators? 3) Does this protein have a role in interaction of JNK and c-Jun? There may be two possibilities; 1) WDR13 may be involved in exchange of co-repressors with co-activators in the presence of JNK signal by proteosomal degradation of co-repressors at AP1 sites or 2) WDR13 may act like an adaptor for c-Jun, which in turn helps in both repression and activation of c-Jun in a signal-dependent manner. At present we do not understand which one of the above possibilities exists. However, interaction of c-Jun and JNK is neither dependent upon JNK catalytic activity nor only on the presence of JNK target sites in c-Jun [33]. It has been suggested that JNK in its inactive form contributes to the repressor function [27, 34]. In another study, understanding role of *Wdr13* gene in the brain tissues, we have observed activation of some of the AP1 target genes in *Wdr13* ^*-/0*^ null mice [35] and in present study higher basal AP1 reporter activity in *Wdr13* ^*-/0*^ cells and amelioration of AP1 reporter activity after JNK activation suggest that WDR13 is possibly working as c-Jun adapter and may be helping c-Jun/JNK/NCoR1/HDAC3 interactions. However, this needs further validation.

Our previous results in pancreatic beta cells showed that WDR13 binds at p21 promoter and regulates its transcription in MIN6 cells. Since the p21 promoter does not contain an AP1 site, it is possible that binding of WDR13 and c-Jun complex at p21 promoter may be indirectly mediated through sp1 site as suggested by Kardassis et al., [36] or by some other nuclear receptors. In agreement with these results, JNK activation, either by UV in MIN6 and HEK293 cells (Additional file 3: Figure S2C, E) or by anisomycin in MEFs, did not show activation of p21 reporter after WDR13 over-expression (Additional file 3: Figure S2A). These results suggest that regulation of p21 by WDR13 is not through AP1, but through some other mechanism that may be cell type-specific and is not yet understood. We have provided evidence that WDR13 protein is part of the repressor complex NCoR1/HDAC3. In the presence of JNK signal this protein acts as a transcriptional activator of AP1 target genes. The lack of evidence of interactions of WDR13 with either NFκB and/or SMRT in our study showed that WDR13 might be exclusively associated with c-Jun and NCoR complex to regulate AP1 target gene transcription.

The role of c-Jun in cell proliferation and malignancy has been shown in many tissues including liver [26], mammary glands [37] and colon [29, 38]. However, the role of c-Jun in apoptosis has been shown to differ in various cell types. Some studies suggest that phosphorylation at serine 63/73 of c-Jun protects cells from apoptosis [39], whereas others suggest that phosphorylation of c-Jun supports apoptosis [40]. WDR13 activates c-Jun activity in JNK-dependent manner, and the lack of WDR13 attenuates c-Jun phosphorylation and its activity. Our results are in agreement with the protective role of c-Jun phosphorylation in apoptosis. The expression of *Wdr13* in colon along with the well-known function of c-Jun in the development of colorectal tumor encouraged us to analyze the phenotype of *Wdr13* knockout mice. There was an increase in expression of AP1 target genes in *Wdr13* ^*+/0*^ mice in the proximal colon as compared to control mice after AOM/DSS treatment (Fig. 7). Interestingly, after DSS treatment, *Wdr13* ^*-/0*^ mice showed more ulceration (Fig. 2f) than wild-type littermates, which may explain the reduced incidence of colitis-induced colorectal tumor in these mice. Other studies have suggested that the increased apoptosis may lead to increase in ulceration [41]. It is the increased levels of p-JNK in AOM/DSS model that causes induction of AP1 target genes such as *lgr5* and *cd44* in *Wdr13* ^*+/0*^ mice as compared to non-treated controls [18]. However, there was no activation of AP1 target genes (Fig. 7), and protection from AOM/DSS-induced colorectal tumor in *Wdr13* ^*-/0*^ mice. Various AP1 target genes are regulated by both canonical and non-canonical Wnt signalling due to cross talk among these pathways [9]. In the present study, we did not detect any interaction of WDR13 with β-catenin (Fig. 6c). These results suggest that the regulation of AP1 target genes by WDR13 is less likely to be through canonical Wnt signalling. However, further experimentation is needed to rule out the involvement of canonical Wnt signalling in the regulation of AP1 target genes in *Wdr13* null mice. Some of the AP1 target genes are also regulated by NFκB transcription factor [16], and inactivation of IKKβ - an activator of NFκB in intestinal epithelial cells - leads to dramatic reduction in tumor number after AOM/DSS treatment [41] due to increase in apoptosis. The reduced expression of *lgr5* [42] and *cd44* [43] in *Wdr13* ^*-/0*^ mice may be another factor responsible for the increase in apoptosis. Surprisingly, in spite of the reduced levels of proliferative markers such as *lgr5* and *cd44* in *Wdr13* ^*-/0*^ mice after AOM/DSS treatment, there was no decrease in the number of BrdU positive cells in crypts of colon. Since inflammation and tumorigenesis are inter-linked, and in this study we used whole body knockout mice, we cannot rule out the involvement of immune cells in reduction of colitis-induced colorectal cancer in these mice. Consistent with these results the reduced inflammation on high fat diet or in diabetic mouse model background in *Wdr13* ^*-/0*^ mice may contribute to the increased islet mass and increased beta cell proliferation in pancreas along with the reduced levels of p21 [22, 44].

## Conclusions

In conclusion, we provide evidence that WDR13 is a novel transcriptional activator of AP1 target genes in the presence of JNK signal. The lack of *Wdr13* gene conferred protection to a large extent against colitis-induced colorectal tumor in mice through reduced expression of AP1 target genes. Given the expression of WDR13 in various cell types of colon, cell-specific knockout of this gene will further help to explain the phenotype in detail.

## Additional files

Additional file 1: Table S1. Primer list for real time PCR. (DOC 15.5 kb)
Additional file 2: Figure S1. Cell growth, cell cycle and apoptosis analysis of *Wdr13* ^+/0^ and *Wdr13* ^-/0^ MEFs at passage 3. A. Growth curve of *Wdr13* ^+/0^ and *Wdr13* ^-/0^ MEFs at passage 3 (*n* = 3). B. Cell cycle analysis of unsynchronized MEFs. C. Annexin V staining of MEFs. n - Shows number of independently derived MEFs used in respective experiments for *Wdr13* ^+/0^ and *Wdr13* ^-/0^ genotypes. (TIF 4700 kb)
Additional file 3: Figure S2. Effect of WDR13 on p21 and c-Jun reporter assay in various cell lines. A. p21 promoter activity in MEFs before and after 4 h treatment of anisomycin. Black bar, *Wdr13* ^+/0^ MEFs; white bar, *Wdr13* ^-/0^ MEFs; Light grey bar, MEFs overexpressing WDR13. (p21-Luc- 350 ng, β-gal-100 ng, pGFP-150 ng or pCMV-FLAG-*Wdr13*-150 ng) B. c-Jun reporter activity in MIN6 cells 6 h after UV treatment. C. p21 reporter activity in MIN6 cells before and 6 h after UV treatment. D. c-Jun reporter activity in HEK293 cells 6 h after UV treatment. E. p21 reporter activity in HEK293 cells before and 6 h after UV treatment. (For panel B-E, c-Jun-Luc or p21-Luc- 350 ng, β-gal-100 ng, pGFP-150 ng or pCMV-FLAG-*Wdr13*-150 ng). All transfections were performed in technical triplicates. (TIF 5959 kb)

## Abbreviations

AOM: Azoxymethane
AP: Activator protein
CCMB: Centre for cellular and molecular biology
DSS: Dextran sodium sulphate
H&E: Haematoxylin and eosin
HRP: Horseradish peroxidase
IKKβ: Nuclear factor kappa-B kinase subunit beta
JNK: c-Jun N-terminal kinase
LGR5: Leucine-rich repeat-containing G-protein coupled receptor 5
MEF: Mouse embryonic fibroblasts
NFκB: Nuclear factor kappa-light-chain-enhancer of activated B cells
qRT-PCR: Quantitative RT-PCR
WDR13: WD repeat domain 13
