# Supplementary material for: WD-repeat protein WDR13 is a novel transcriptional regulator of c-Jun and modulates intestinal homeostasis in mice
Source: BMC Cancer. 2017 Feb 21;17:148. doi: 10.1186/s12885-017-3118-7 (PMC5320654; doi:10.1186/s12885-017-3118-7)
Supplement: Additional file 1: Table S1. — Primer list for real time PCR. (DOC 15.5 kb) [file 12885_2017_3118_MOESM1_ESM.doc]

| Additional file 1: Table S1 Primer list for real time PCR |
| --- |
| *CD44* : 5’- CTCCTGGCACTGGCTCTGA-3’ *CD44* :  5’-CTGCCCACACCTTCTCCTACTATT-3’ |
| *CCDN1*:  5’-GTGCGTGCAGAAGGAGATTGT -3’ *CCDN1*:  5’-CTCACAGACCTCCAGCATCCA-3’ |
| *AXIN2*:  5’-GGTTCCGGCTATGTCTTTGC-3’ *AXIN2*:  5’-CAGTGCGTCGCTGGATAACTC-3’ |
| *TCF7L2*: 5’-GAGAGTGCAGCCATCAACCAG-3’  *TCF7L2*: 5’-GTGATCGGAGGAAGCGAAAGG -3’ |
| *GRP49*:  5’-CGGAGGAAGCGCTACAGAAT-3’ *GRP49*:  5’-CTGGGTGGCACGTAGCTGAT-3’ |
| gapdh: 5’-ACCCAGAAGACTGTGGATGG-3’  gapdh: 5’-CACATTGGGGGTAGGAACAC-3’ |
