# Supplementary figures and images for: WD-repeat protein WDR13 is a novel transcriptional regulator of c-Jun and modulates intestinal homeostasis in mice
Source: BMC Cancer. 2017 Feb 21;17:148. doi: 10.1186/s12885-017-3118-7 (PMC5320654; doi:10.1186/s12885-017-3118-7)

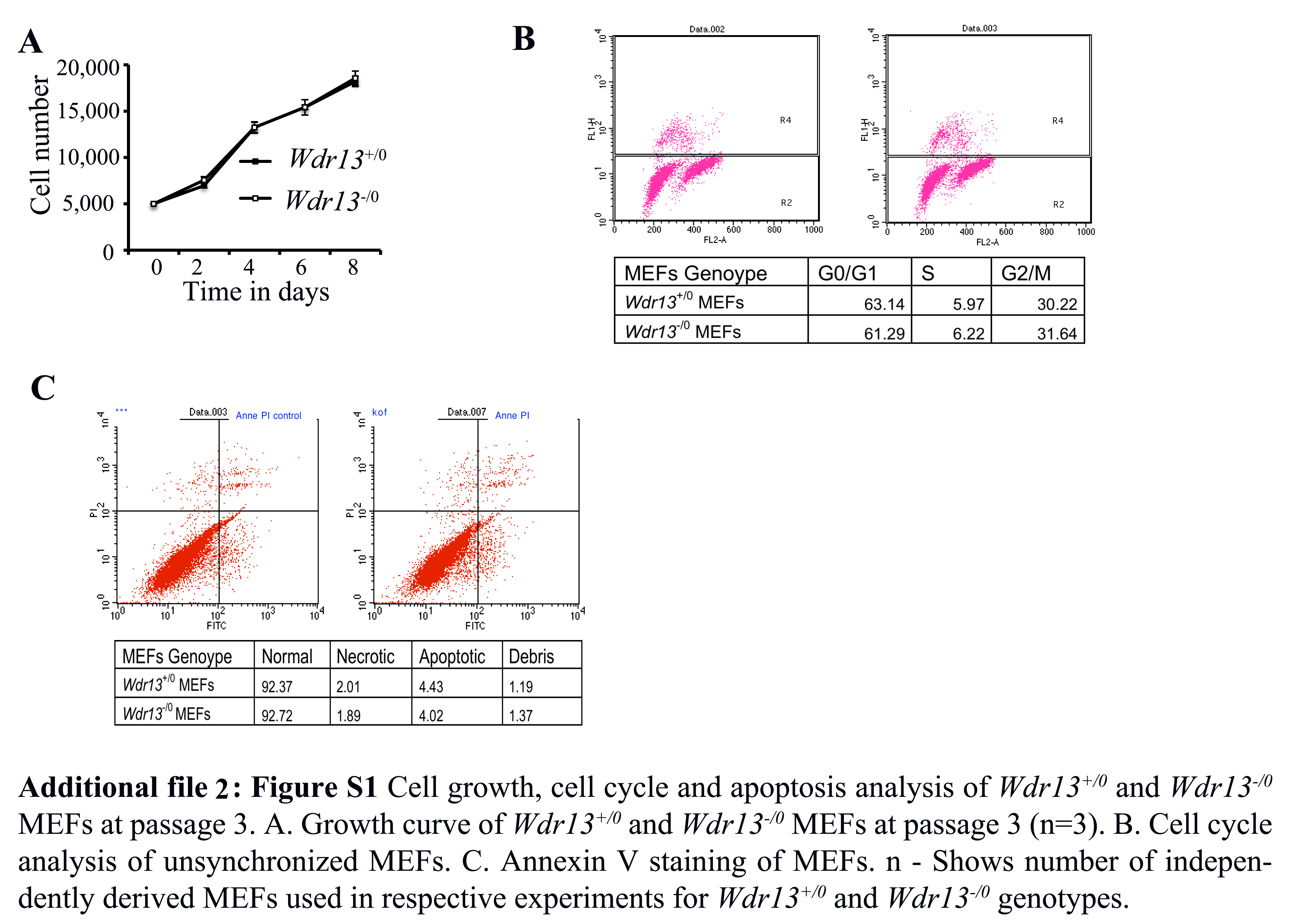

Supplement: Additional file 2: Figure S1. — Cell growth, cell cycle and apoptosis analysis of Wdr13 +/0 and Wdr13 -/0 MEFs at passage 3. A. Growth curve of Wdr13 +/0 and Wdr13 -/0 MEFs at passage 3 (n = 3). B. Cell cycle analysis of unsynchronized MEFs. C. Annexin V staining of MEFs. n - Shows number of independently derived MEFs used in respective experiments for Wdr13 +/0 and Wdr13 -/0 genotypes. (TIF 4700 kb) [file 12885_2017_3118_MOESM2_ESM.tif]

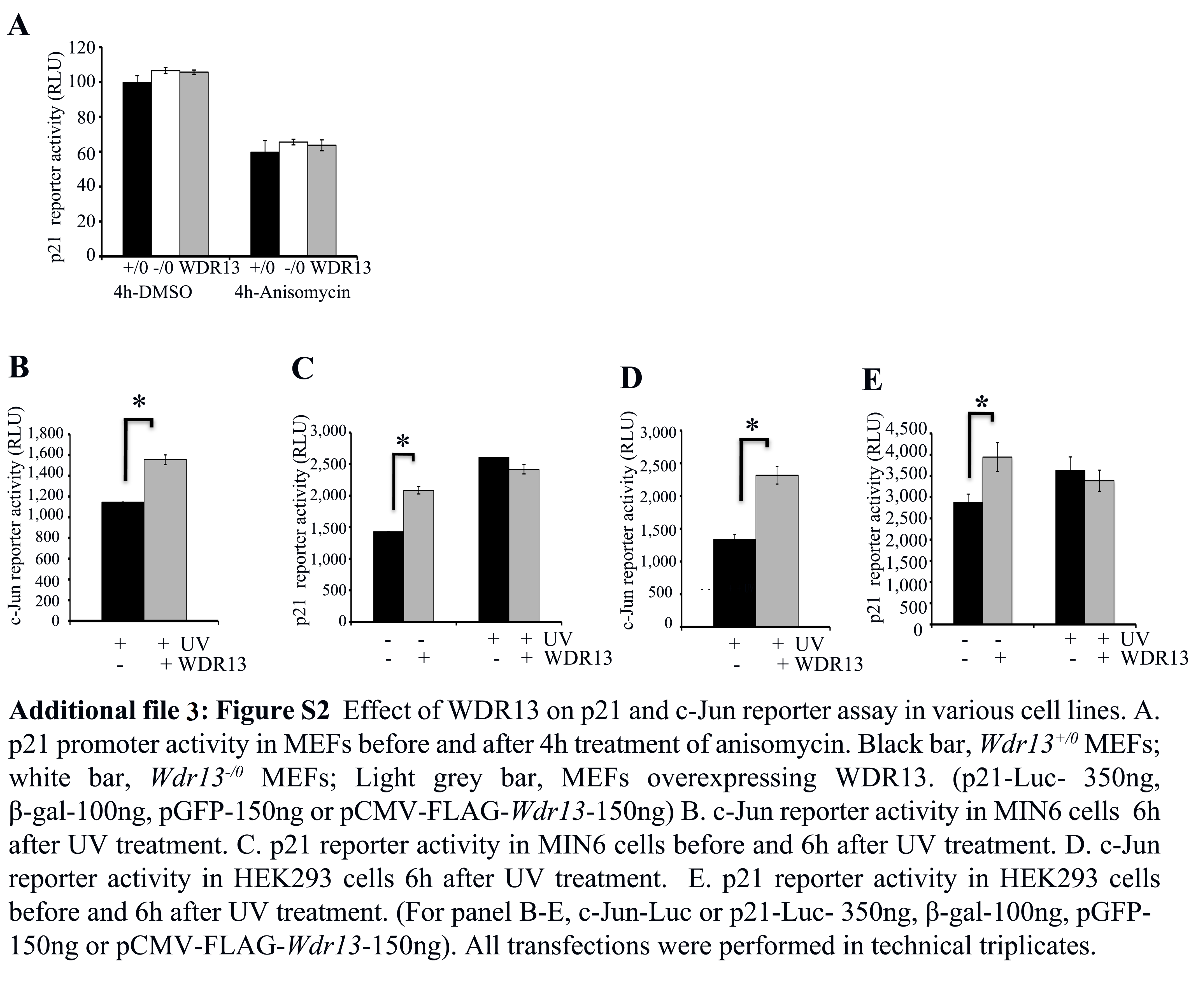

Supplement: Additional file 3: Figure S2. — Effect of WDR13 on p21 and c-Jun reporter assay in various cell lines. A. p21 promoter activity in MEFs before and after 4 h treatment of anisomycin. Black bar, Wdr13 +/0 MEFs; white bar, Wdr13 -/0 MEFs; Light grey bar, MEFs overexpressing WDR13. (p21-Luc- 350 ng, β-gal-100 ng, pGFP-150 ng or pCMV-FLAG-Wdr13-150 ng) B. c-Jun reporter activity in MIN6 cells 6 h after UV treatment. C. p21 reporter activity in MIN6 cells before and 6 h after UV treatment. D. c-Jun reporter activity in HEK293 cells 6 h after UV treatment. E. p21 reporter activity in HEK293 cells before and 6 h after UV treatment. (For panel B-E, c-Jun-Luc or p21-Luc- 350 ng, β-gal-100 ng, pGFP-150 ng or pCMV-FLAG-Wdr13-150 ng). All transfections were performed in technical triplicates. (TIF 5959 kb) [file 12885_2017_3118_MOESM3_ESM.tif]
